# Homopeptide and homocodon levels across fungi are coupled to GC/AT-bias and intrinsic disorder, with unique behaviours for some amino acids

**DOI:** 10.1038/s41598-021-89650-1

**Published:** 2021-05-11

**Authors:** Yue Wang, Paul M. Harrison

**Affiliations:** Department of Biology, McGill University, Montreal, QC Canada

**Keywords:** Molecular evolution, Intrinsically disordered proteins, Computational biology and bioinformatics, Protein analysis, Proteome informatics, Sequence annotation

## Abstract

Homopeptides (runs of one amino-acid type) are evolutionarily important since they are prone to expand/contract during DNA replication, recombination and repair. To gain insight into the genomic/proteomic traits driving their variation, we analyzed how homopeptides and homocodons (which are pure codon repeats) vary across 405 *Dikarya*, and probed their linkage to genome GC/AT bias and other factors. We find that amino-acid homopeptide frequencies vary diversely between clades, with the AT-rich *Saccharomycotina* trending distinctly. As organisms evolve, homocodon and homopeptide numbers are majorly coupled to GC/AT-bias, exhibiting a bi-furcated correlation with degree of AT- or GC-bias. Mid-GC/AT genomes tend to have markedly fewer simply because they are mid-GC/AT. Despite these trends, homopeptides tend to be GC-biased relative to other parts of coding sequences, even in AT-rich organisms, indicating they absorb AT bias less or are inherently more GC-rich. The most frequent and most variable homopeptide amino acids favour intrinsic disorder, and there are an opposing correlation and anti-correlation *versus* homopeptide levels for intrinsic disorder and structured-domain content respectively. Specific homopeptides show unique behaviours that we suggest are linked to inherent slippage probabilities during DNA replication and recombination, such as poly-glutamine, which is an evolutionarily very variable homopeptide with a codon repertoire unbiased for GC/AT, and poly-lysine whose homocodons are overwhelmingly made from the codon AAG.

## Introduction

Homopeptides and homocodons (which are perfect codon repeats) are well known for their roles in inherited human diseases, such as poly-CAG/poly-Gln in Huntington’s disease, and poly-Ala linked to congenital developmental disorders^1^. The pathogenic mechanisms of these diseases are various. While many diseases might be essentially caused by the aggregation propensity of some homopeptide types^2, 3^, the soluble forms of proteins with longer mutant repeats could also be problematic by competing with functional homopeptides in normal proteins for molecular interactions^4^. Homopeptides and homocodons not exceeding certain lengths are prevalent and can be beneficial for eukaryotes^5^. About 15% of proteins in any eukaryotic proteome contain at least one stretch of ≥ 5 identical residues^6^. These homopeptide-containing proteins function diversely, especially in DNA/RNA binding, signaling and regulation^7–9^. Homopeptides levels generally exceed those of other amino-acid repeat types^10^.

Nevertheless, the functions of prevalent homopeptides or homocodons are still largely unclear, and most might not be essential but rather create diversity in genomes which can be selected on^11^. Homopeptide lengths are often polymorphic between different individuals in a species, and even between different cell types or at different organismal ages^12, 13^. Although phenotypic evolution is mostly modulated by *cis*-regulatory elements, homopeptide length polymorphisms are also linked to significant morphological differences, e.g., in dogs^14^. Homopeptide length variations are proposed as a ‘tuning knob’ that acts through expansion and contraction between generations, enabling greater phenotypic variability in a population^11^. Besides the high mutation rate of homopeptides themselves, DNA substitution rate is also strongly correlated with the distance to homopeptides, and also insertions/deletions are frequently associated with homopeptides in their flanks^15, 16^. Thus, homopeptides may enable rapid protein divergence, through creating more polymorphism.

Early studies found that eukaryotes have unique homopeptide distributions, i.e., their proteomes prefer/tolerate homopeptides at different lengths for different amino acids^17^. It was suggested that amino-acid preferences in low-complexity regions or homopeptides are largely driven by bias in genomic AT (adenine + thymidine) or GC (guanidine + cytidine), and are under selection pressures^16, 18^. Also, previous analyses have shown that homopeptides are enriched in intrinsically disordered regions (IDRs)^19–22^, as are tandem repeats generally^10, 19, 21^.

Mularoni, et al*.* examined tandem repeat evolution across 12 vertebrate species, and by comparing to noncoding DNA repeats inferred that there is selection maintaining prevalent tandem repeats^23^. Schaper, et al*.* discovered that ~ 60% of tandem-repeat regions are deeply conserved as such across 61 eukaryotes^24^. A few other studies have focused on homopeptide evolution. In a comparison of 13 diverse eukaryotes, homopeptides were found to have no general GC- or AT -bias, and homocodons within homopeptides were longer than expected by chance^7^. Across five eukaryotes, homopeptides were enriched inside alternatively-spliced exons, which also had longer homocodons and lower codon diversity^18^. In a study of > 600 human genes, homopeptide tracts had relatively elevated mutation rates^22^. Mier, et al. discovered different positional trends for homopeptides made from different amino-acid types for a diverse sample of cellular organisms^25^. Distinct trends in conservation of compositional biases for different amino-acid types in annotated IDRs were observed in a survey of > 10,000 proteomes^26^.

Previously, it was observed that a large-scale emergence of prion-like regions during *Saccharomycetes* yeast evolution was caused by mutational trends that produced more poly-asparagine tracts^27^. Motivated by these findings, we hypothesized that the factors driving the evolution and variation of homopeptides and homocodons in general would also be discernible through analysis of their trends across a large diverse fungal clade, i.e., the subkingdom *Dikarya*, comprised of the phyla *Basidiomycota* and *Ascomycota*. Previous studies have not analyzed how the factors underlying homopeptide/homocodon formation influence their variation between clades in a diverse organismal phylogeny in an integrated manner. In this study, we probe in detail how, over hundreds of millions of years of fungal evolution, both homopeptide and homocodon variation are coupled to or modulated by GC/AT bias and intrinsic disorder propensity, and discover some unique behaviours for specific amino acids and codons.

## Results and discussion

The evolutionary behaviour of homopeptides and homocodons (perfect codon repeats) is surveyed across the fungal *Dikarya* sub-kingdom. In this survey, we had the following objectives:

1. To derive an overview of the variation in homopeptide frequencies, identifying any anomalous behaviour in specific clades;
2. To examine how homopeptide frequencies are influenced by or coupled to genomic AT/GC bias, which is the most basic compositional parameter typically studied in such analyses;
3. To examine how codon preferences in homocodons and homopeptides are affected by such AT/GC bias, in doing so deriving a measure of homopeptide purity (i.e., the predominance of one specific codon in homopeptides);
4. To examine how proteomic homopeptide frequencies are influenced by intrinsic disorder and structured domain content in proteins.

### Homopeptide levels vary extensively across diverse fungi

**Figure 1:**
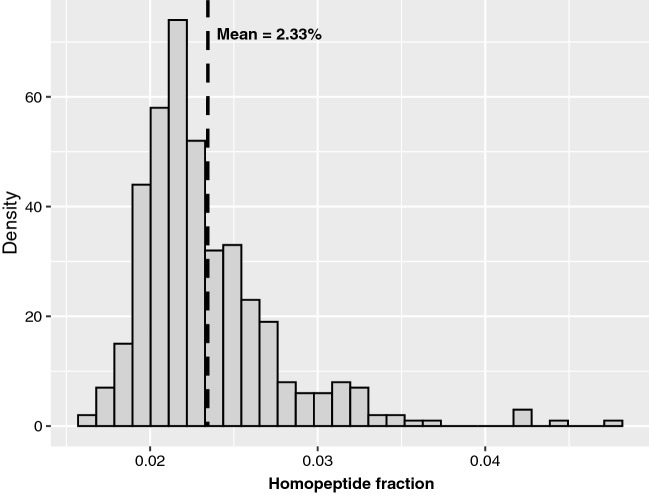
Distribution of overall homopeptide fraction in the proteomes. Mean = 0.023, standard deviation = 0.004, skewness = 2.004, kurtosis = 9.350. Each bin is 0.01 long and labelled with its lower bound.

The distribution of homopeptide frequencies (1.64–4.78%) in the 405 proteomes of *Dikarya* shows a heavy-tailed right-skewed distribution. Nearly 70% of values are in the small range 1.8–2.4%. Only a few proteomes have homopeptide frequencies below this range, the rest varying from 2.4 to 4.8% (Fig. 1). Thus, while most proteomes have similar homopeptide fractions, there is a bias towards homopeptide accumulation for values away from this peak.

**Figure 2:**
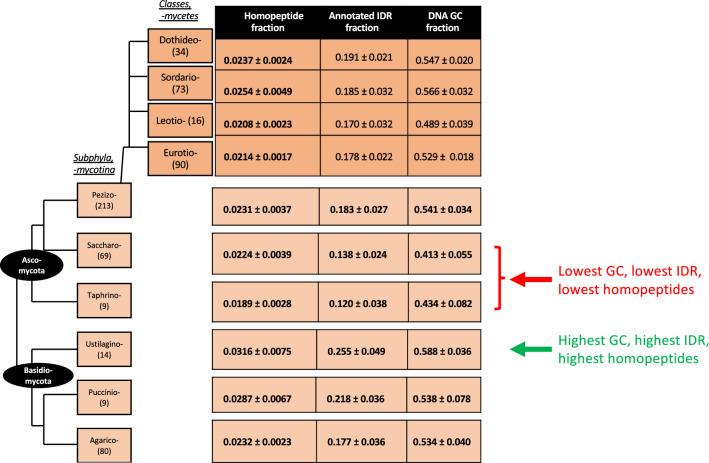
Schematic *Dikarya* phylogenetic tree with mean fractions of homopeptides, annotated IDRs and DNA GC content. The values for sub-phyla (clades suffixed ‘-mycotina’), and classes (suffixed ‘-mycetes’) within large subphylum *Pezizomycotina* are shown.

We examined the trends in homopeptide frequencies across 405 *Dikarya* (comprising the phyla *Basidiomycota* and *Ascomycota*), and also examined other various attributes, including GC content and annotated IDR content (Fig. 2 and Suppl. Figure S1). Subphyla (and classes within the large subphylum *Pezizomycotina*) are analyzed in Fig. 2, with details of species names and prevalent amino-acid / codon types in Suppl. Figure S1. Fractions of homopeptides and IDRs are colour-coded by spectra in Suppl. Figure S1. The lowest homopeptide fractions are for *Saccharomycotina* and *Taphrinomycotina*, which are also low-GC and have the lowest annotated IDR fractions (Fig. 2). Variation of homopeptide fractions is obvious between different clades, but homopeptides and annotated IDRs can also accumulate in specific species over a short evolutionary time (Suppl. Figure S1, sections *a-b;* lighter colours for higher fractions).

Heat maps of the most abundant homopeptides and homocodons (i.e., perfect codon repeats) were derived (Suppl. Figure S1, sections *c* and *d*). The key to these heatmaps is supplied with the legend to Suppl. Fig. S1. To show which homopeptides and homocodons predominate, they are ranked in decreasing order of overall frequency (i.e., total fraction of amino acids or codons of that type) in each proteome. Homopeptide and homocodon length distributions are characterised using slopes from log–log plots as described in “Methods” section. For these length distributions, lighter colours in heat map cells indicate more small homopeptides or homocodons, and darker colours a greater amount of long ones. One can see that generally there are more lighter cells for sections *c* and *d* (shorter homopeptides and homocodons) where the overall homopeptide fraction is lower (darker in section *a*) (Suppl. Figure S1). When we examine the relationship between the log(length) distribution slopes and corresponding homopeptide frequencies for each amino acid, we see that there are statistically significant correlations for most amino-acid types, although all but two have weak coefficients < 0.3 (Suppl. Figure S2). These results suggest that a tendency to shorter homopeptide sizes contributes in some way to there being fewer homopeptides in a proteome, or vice versa.

The frequency ranking of homopeptides of different amino-acid types can also change within smaller clades and genera (Suppl. Figure S1). Such changes even appear between strains of one species. For example, among six strains of yeast *Saccharomyces cerevisiae*, most of the top ten homopeptides shift frequency ranking compared to other strains. Homopeptide lengths for aliphatic hydrophobic residues, i.e., poly-Leu, poly-Ile, poly-Val, are generally short across all *Dikarya* (lighter cells in Suppl. Figure S1 heat maps), possibly due to selection against protein aggregation^17^, and constraints of side-chain packing in protein-domain hydrophobic cores.

The amino acids that vary the most in homopeptide amount are discerned from examining the standard deviations for their ranking for homopeptide frequencies (Table 1). The top one third of the homopeptides that change the most across *Dikarya* are especially highlighted in red in Table 1 (‘Rank standard deviation’ column). All but one of these are from amino acids whose codon repertoire is biased for GC or AT (Table 1). However, poly-Gln specifically stands out as encoded by a codon repertoire that has no overall GC/AT-bias, but it still greatly changes in the frequency ranks across *Dikarya* (Table 1).

**Table 1:**
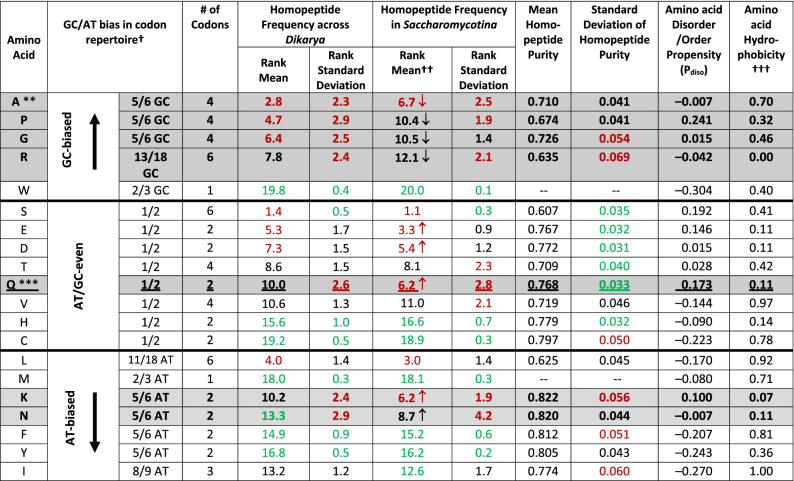
Amino-acid homopeptide frequency ranks and purities, disorder propensities and hydrophobicities

Amino acids are put in the order from the highest GC bias to the highest AT bias of their encoding codons, and if at the same bias level, they are in the order of the homopeptide frequency rank mean. The frequency rank mean is the mean of the ranking for the amino acid according to its frequency in homopeptides in a proteome; also listed are the rank standard deviations. These are calculated both across the whole *Dikarya* set and just within *Saccharomycotina*.

**Coloured red are the top thirds of the list of amino acids sorted on: homopeptide frequency rank mean and standard deviation across *Dikarya* and within *Saccharomycotina*, and standard deviation of homopeptide purity. Similarly, the bottom thirds of these lists are coloured green. Also, the rows in the table for amino acids with homopeptide frequency rank deviation across *Dikarya* in the top one third of amino acids are in bold and shaded.

*** Q (glutamine) is underlined since among amino acids with an AT/GC-even codon repertoire, the rank of poly-Q is the most variable across *Dikarya*.

^†^GC/AT bias is the fraction of AT or GC in the encoding codon repertoire for an amino acid.

^††^Amino acids that rise or fall by > or < 1.0 ranking place on average in *Saccharomycotina*, compared to *Dikarya* generally, are labelled with up or down arrows respectively.

^†††^Kyte-Doolittle hydrophobicity scale normalized to the interval 0.0 to 1.0.

### *Saccharomycotina* have distinct behaviour for homopeptide and homocodon evolution

Previous work on limited data sets indicated that the prevalent types of homopeptides are influenced by GC bias, and high GC content is linked to homopeptide formation^28–32^. Here, we investigated the effect of GC/AT levels on homopeptide and homocodon evolution on a large scale across *Dikarya*, and for *Saccharomycotina* in particular. *Saccharomycotina* are mostly AT-rich while species in other subphyla are mostly GC-rich, which causes homopeptide composition in *Saccharomycotina* to be distinct (Suppl. Figure S1, section c). The four homopeptide types which drop most in the frequency ranks in *Saccharomycotina* are all for GC-rich amino acids (Table 1), while the two types that rise the most in rank are poly-Asn and poly-Lys, which have AT-rich codons (Suppl. Figure S1; Table 1). This result concurs with the discovery in analyses of prion-like proteins in *Saccharomycotina* that GC% influences the abundance of compositionally-biased protein regions encoded by GC- or AT-rich codons^27, 33^.

Given that homopeptides behave differently in the AT-rich *Saccharomycotina* relative to other subphyla, we investigated more closely how homopeptide and GC/AT trends are related.

### Homopeptides tend to be GC-rich even for AT-rich genomes

**Figure 3:**
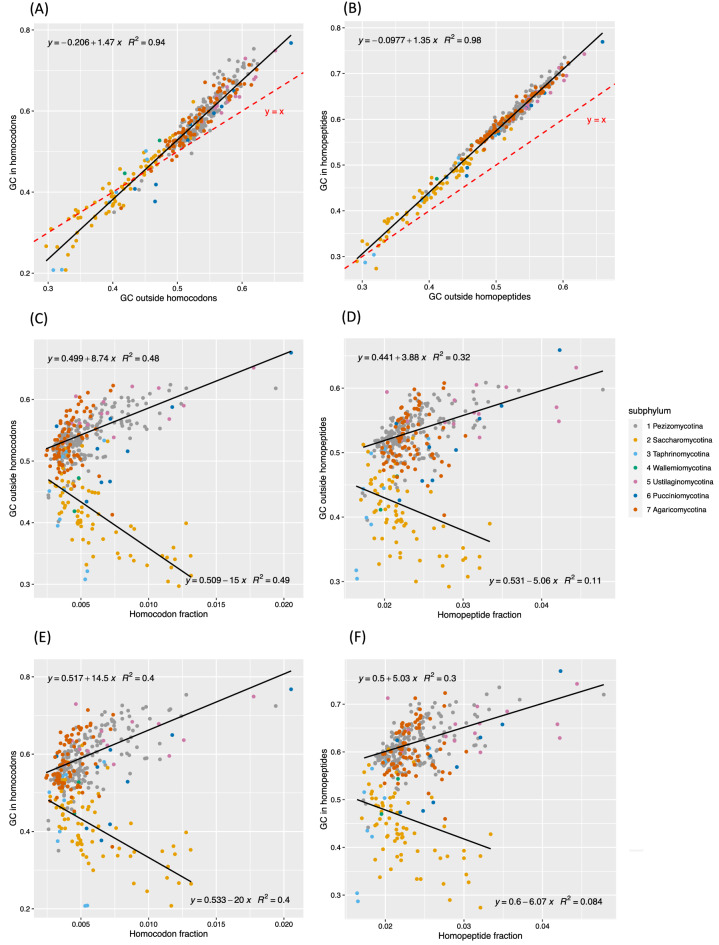
Relationship between homopeptide/homocodon level and GC/AT level. (**a**) GC/AT level in homocodons versus outside homocodons in coding regions. The red dashed line shows the default where GC/AT levels outside and inside homocodons are identical (*y* = *x* line). (**b**) GC/AT level in homopeptides versus outside homopeptides. The *y* = *x* line is shown (red dashed line). (**c**) GC/AT-level outside homocodons versus the fraction of homocodons, with separate linear regressions for GC-biased and AT-biased organisms. That is, they are separated into two groups one with GC fraction ≥ 0.5, and one with GC fraction < 0.5. (**d**) GC/AT-level outside homopeptides versus the fraction of homopeptides, with separate linear regressions for GC-biased and AT-biased organisms, as in part (**c**). (**e**) GC/AT-level in homocodons plotted versus the fraction of homocodons, with separate linear regressions for GC-biased and AT-biased organisms, as in part (c). (**f**) GC/AT-level in homopeptides plotted versus the fraction of homopeptides, with separate linear regressions for GC-biased and AT-biased organisms, as in part (**c**). All correlations in parts (**a**)–(**f**) are significant at *P* < 0.05. A legend explaining the colour-coding for each subphylum is at the right of the figure.

It is obviously expected that the AT/GC level in coding regions outside of homopeptides/homocodons and within them are positively correlated to each other (Fig. 3a-b). To examine how different are the AT/GC levels within and outside homopeptides/homocodons, we examined how the linear regressions deviate from the *y* = *x* line for both homopeptides and homocodons. GC level tends to be higher within homocodons in both AT- and GC-rich organisms, but for a large fraction of AT-rich (GC-poor) species, homocodons are more AT-rich than other proteome areas (Fig. 3a). For homopeptides, however, there is an underlying GC bias relative to outside of homopeptides even in AT-rich (GC-poor) organisms (i.e., mainly the *Saccharomycotina*) (Fig. 3b). This is also evident in Table 1, where only one of the top ten overall most frequent amino acids in homopeptides has an AT-biased codon repertoire, but five of them have a GC-biased codon repertoire. This may be because GC level is easier to increase in homocodons/homopeptides than AT level. Pathogenic GC-rich homocodons such as CAG/GTC and CGG/GCC, are found to be particularly prone to expand in models and in experiments, with a higher inherent slippage rate which is determined by propensity to form stable mismatched secondary structures^34–36^. The two repeats (CAG and CGG) are able to encode seven frequent homopeptide amino acids including Gln, Ser, Ala, etc*.*, since reading frame should not affect the inherent slippage rate. Also, GC-rich low-complexity regions (including homopeptides) are recombination hotspots which may lead to increased homopeptide content^37^.

Given these trends, we investigated the relationship between homocodon/homopeptide levels and GC- or AT-bias across *Dikarya*.

### Homocodon/homopeptide accumulation is strongly coupled to GC/AT bias, with a bi-furcated correlation arising between homocodon/homopeptide levels and GC/AT bias

We probed the relationship between homopeptide and homocodon levels and GC/AT bias, across proteomes (Fig. 3c–f). Interestingly, the correlation between homocodon fraction and AT/GC content splits into two directions from around 50% AT/GC (Fig. 3c–d, with linear regressions fitted to the data split into AT-biased and GC-biased groups). This indicates that homocodon abundance is positively correlated with the extremeness of AT/GC bias. Also, homocodon levels are lower for species that tend to mid-GC (~ 50% GC). Such a correlation is less strong for homopeptides but still significant (Fig. 3e–f). We would expect there to be no major bars on homocodon formation simply because a genome has medium GC/AT levels. Thus, general selection pressures or mutational biases governing GC/AT bias are majorly coupled to homocodon formation and also strongly influence the appearance of homopeptides.

The factors leading to the variation of genomic GC level during evolution are complicated, including both mutational bias and natural selection^38^. When the global GC content switches due to events such as horizontal gene transfer and biased gene conversion, the concentrations of tRNA with different anticodons could quickly readjust to fit the new GC level, which would further drive the shift in codon-usage bias gradually from current abundant codons to new optimal codons^39–41^. The decrease of concentrations of the previously optimal tRNAs could induce selective pressure or point mutations in previous optimal homocodons, since homocodons demanding previous tRNAs would slow down translation^42^. Also, the increase of the new optimal tRNA could influence expansion of corresponding homocodons. On the other hand, homopeptide expansion is an efficient way to increase local GC or AT bias, and point mutation rates are also higher in homopeptides, since they are generally located in regions under less constraint, which both lead to faster GC level change, to be further selected on by the changed tRNA concentrations^42^. AT/GC-biased regions also naturally accumulate homocodons more easily due to a higher possibility of the same codons co-occurring within a biased region.

The results here imply that general selection pressures or mutational biases governing GC or AT bias influence homocodon/homopeptide levels. The opposite causation, i.e., that homocodon levels are driving GC/AT bias, is not likely since homocodons are such a small fraction of proteomes, although there may be a degree of feedback as newly-formed homocodons accumulate mutations. Despite this link, homopeptides tend to be more GC-rich than other areas of proteomes, even in AT-rich organisms, indicating they absorb AT bias trends less than other areas of the proteome, or have an inherent tendency to higher GC content, as discussed above.

### Homocodon codon preferences correlate with AT/GC bias for some codons, but not for others

It is known that the genomic GC level significantly affects codon usage bias^43–45^, and this is also evident here in the rankings of homocodon frequencies across *Dikarya* (Suppl. Figure S1). To probe this phenomenon, we analyzed the variation in codon preference for the five most common amino acids that are encoded by two alternative codons (E, GAA/GAG; D, GAT/GAC; K, AAG/AAA; N, AAC/AAT; Q, CAG/CAA). Not surprisingly, given the overall trends linked to AT/GC bias discussed above, the codon types in homocodons also change according to the GC/AT-bias of coding regions. The predominant codon encoding poly-Glu in clades of GC-rich species is GAG, but it switches to GAA in the AT-rich *Saccharomycotina* (Suppl. Figure S3). Likewise, the predominant codon encoding poly-Asp switches from GAC to GAT in *Saccharomycotina* (Suppl. Figure S3). Such switching has also been observed for *Drosophila* species^46^.

**Figure 4:**
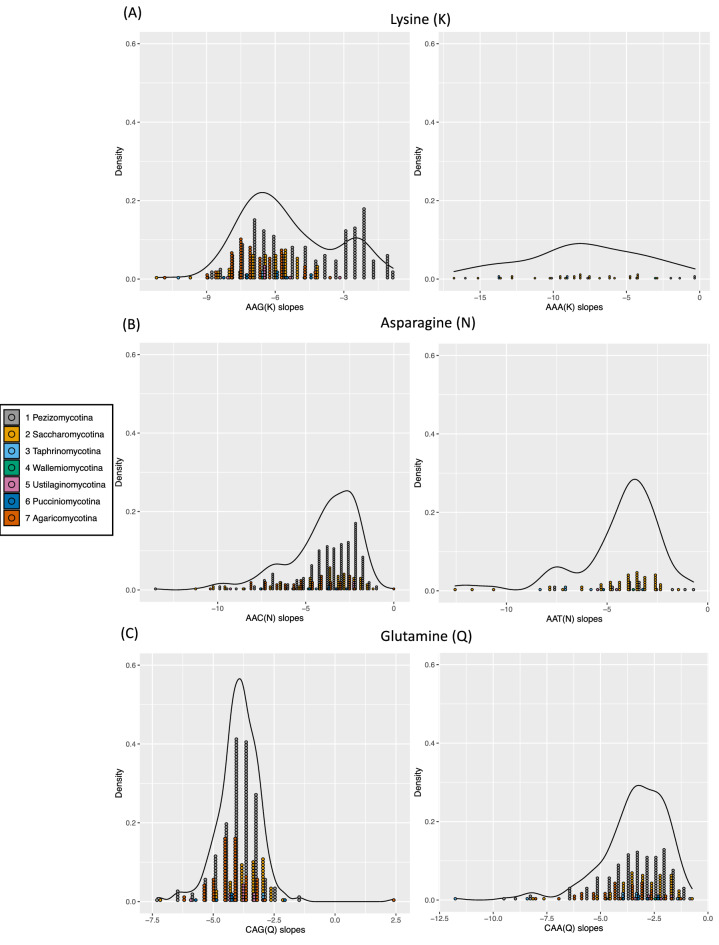
Histograms of length distribution slopes for two synonymous homocodons encoding poly-Lys, poly-Asn and poly-Gln from the top-20 lists of homocodon frequencies. Histograms of the log–log plot slopes for length distributions are plotted (one panel for each synonymous codon). They are binned in intervals of 0.5. For each pair of panels taken together, the total histogram area for each subphylum equals the number of occurrences in the top-20 lists for each homocodon in each subphylum. The lines indicate the overall distribution within each panel. More negative values indicate more, short homopeptides: (**a**) Comparison of Poly-AAG(K), left panel and Poly-AAA(K), right panel; (**b**) Comparison of Poly-AAC(N), left panel and poly-AAT(N), right panel; (**c**) Comparison of Poly-CAG(Q), left panel and poly-CAA(Q), right panel.

To further investigate the effects of AT/GC bias, we examined the log–log plot slopes that indicate the length distributions of homocodons for three different residue types that are encoded by two alternative codons, namely K, N and Q (Fig. 4). Less negative values indicate smaller total relative amounts of short homocodons, and the overall density of the distributions in the different subphyla shows the prevalence of either alternative codon. Each dot in the plots is an occurrence in the top-20 lists of homocodons (arrayed in Suppl. Figure S1). Exceptionally, the predominant codon type for poly-Lys is always AAG, while its synonymous codon AAA only arises a few times in the top 20 frequency ranks even in AT-rich species (Fig. 4a; Suppl. Figure S1). This might be due to selection on poly-Lys at the protein level, and an inherent slippage difficulty for poly-AAA(K) during DNA replication. We focused on trends in the subphyla *Pezizomycotina*,* Saccharomycotina* and *Agaricomycotina*, since they are the largest subphyla*.* Strikingly, the slope distribution for poly-AAG(K) stands out as distinctly bimodal in *Pezizomycotina* (Fig. 4a). This indicates that many species in *Pezizomycotina* have poly-AAG(K) longer than the ordinary length of poly-AAG(K) in other clades. On the other hand, for some amino acids both synonymous homocodons are highly frequent. For example, poly-CAG(Q) and poly-CAA(Q) are both prevalent in *Pezizomycotina* (a GC-rich subphylum) and *Saccharomycotina* (AT-rich), and to a lesser extent *Agaricomycotina* (Fig. 4c). Also, poly-AAC(N) and poly-AAT(N) are both prevalent in *Saccharomycotina* (Fig. 4b; Suppl. Figure S1). This is despite the large-scale mutational trends during *Saccharomycotina* evolution, which have led to more amino acids encoded by more AT-biased codons, and wholesale generation of asparagine-rich regions especially^27^. Generally, these results show that some homocodons have codon preferences that do not follow the overall trends linked to GC/AT content. We surmise that this is due to the inherent slippage probability of specific codons during DNA replication and recombination.

### Purity of homopeptides is modulated by GC/AT bias

Next, we set out to examine the bias of homopeptides for specific codons. To do this, we calculated homopeptide purity. This is defined as the proportion of the most dominant codons in homopeptides, which is influenced by the relative importance of synonymous point mutations *versus* expansions/contractions of homocodons (see “Methods” section). Homopeptide purity was calculated for each amino-acid type (Table 1, Table S1). These amino-acid homopeptide purities vary from clade to clade (Table S1). As explained in the “Methods” section, homopeptide purities will inherently be higher for amino acids with smaller codon repertoires, so we focussed on the standard deviations of purity for analysis. Only 1 of 6 Arg codons is AT-biased, thus although poly-Arg can contain codons with six-fold degeneracy, they can be relatively pure in AT-rich species, most notably *Saccharomycotina* (highlighted red in Table S1; the arginine purity value for *Saccharomycotina* is an outlier). Because of this arginine-specific behaviour, its homopeptide purity varies the most across *Dikarya* (i.e., it has the highest standard deviation of purity, Table 1). In contrast, amino acids that vary the least in homopeptide purity (as evidenced by their overall purity standard deviations, Table 1) have AT/GC-balanced codon repertoires, i.e., equal numbers of A + T and G + C. Thus, homopeptide purity variation is directly related to the GC/AT balance of the codon repertoires of each amino acid.

### Intrinsic disorder is correlated with both homopeptide frequency and variability across *Dikarya*

Homopeptides are prone to accumulate in intrinsically disordered regions (IDRs)^10, 19, 20^. This phenomenon has however yet to be examined evolutionarily across a large phylogeny with many sub-clades with a spectrum of AT- and GC- bias. Thus here, we investigated how homopeptide variation and intrinsic disorder are associated across *Dikarya*.

**Figure 5:**
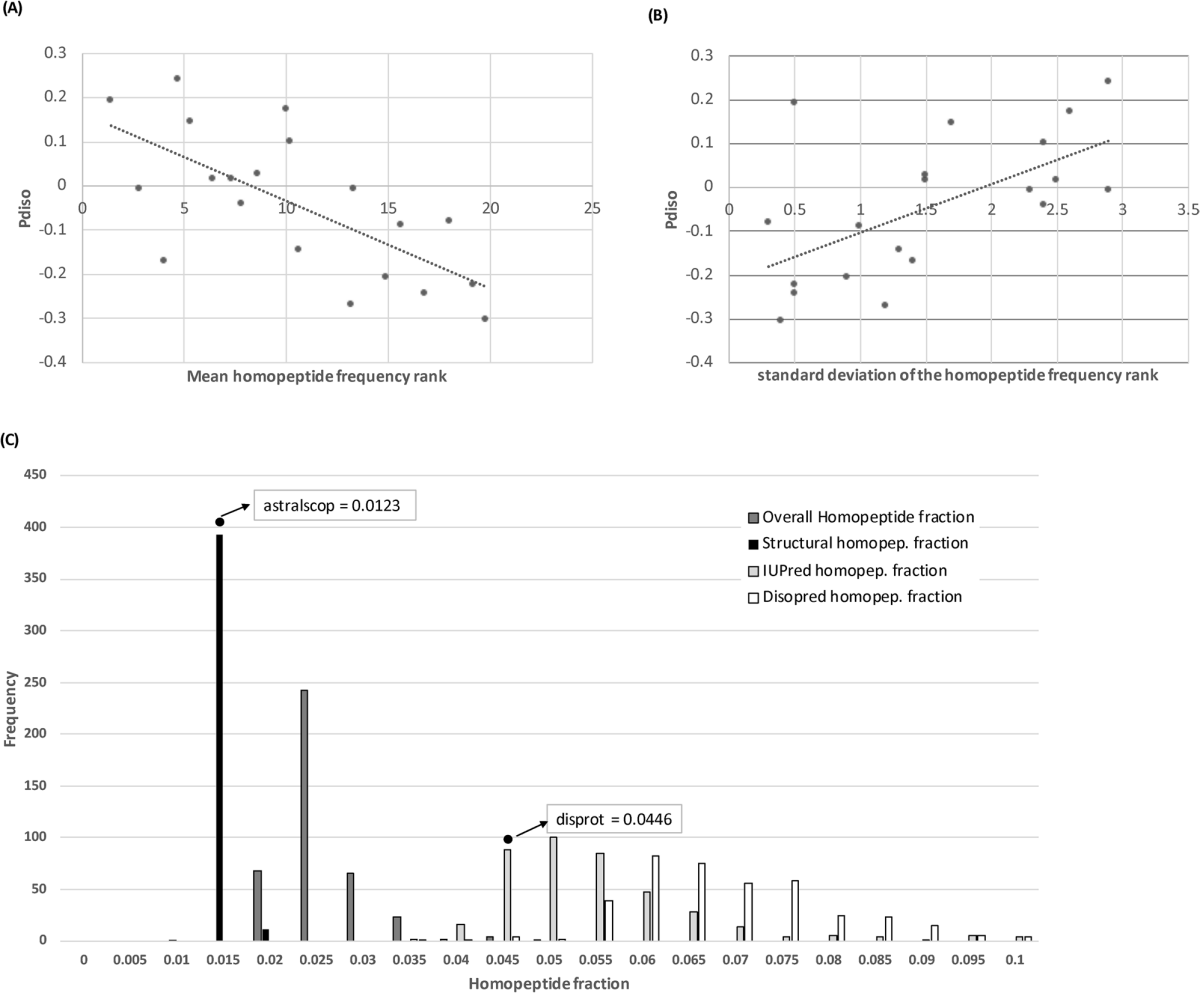
Intrinsic disorder propensity. The intrinsic disorder propensity (**P**_**diso**_ ) of the amino acids is plotted against (**A**) the mean frequency rank across proteomes of the amino acids in homopeptides (Pearson correlation coefficient R = −0.69, *P* = 0.0008), and (**B**) the standard deviation of the frequency rank of the amino acids in homopeptides (R = 0.60, *P* = 0.005). In part (**C**), histograms are depicted of the homopeptide fractions of structured regions (annotations made using SCOP domains), and of the IUPred and DISOPRED intrinsic disorder annotations, with the distribution of the overall homopeptide fractions in the proteomes for comparison. The fractions of homopeptides for disordered regions and for structured regions are calculated as fractions of the total number of residues in the disordered and structured subsets of residues respectively. Also indicated on the plot as points are the homopeptide fractions for the ASTRALSCOP40 and DISPROT databases.

A scale of intrinsic disorder propensity (**P**_**diso**_) was derived from independent data (*Methods*; scale listed in Table 1). We find that **P**_**diso**_ influences both amino-acid frequency (Fig. 5A) and variability (Fig. 5B) in homopeptides across *Dikarya*. Thus, amino acids with higher **P**_**diso**_ vary more from proteome to proteome as homopeptides. Significant correlations are not found for amino-acid hydrophobicities (listed in Table 1). Also, homopeptides are consistently more prevalent in annotated IDRs than in structured domains, and exhibit a far greater variance of frequencies (Fig. 5C). The much narrower variance of homopeptide fractions in structured domains indicates comparatively very tight constraint.

**Figure 6:**
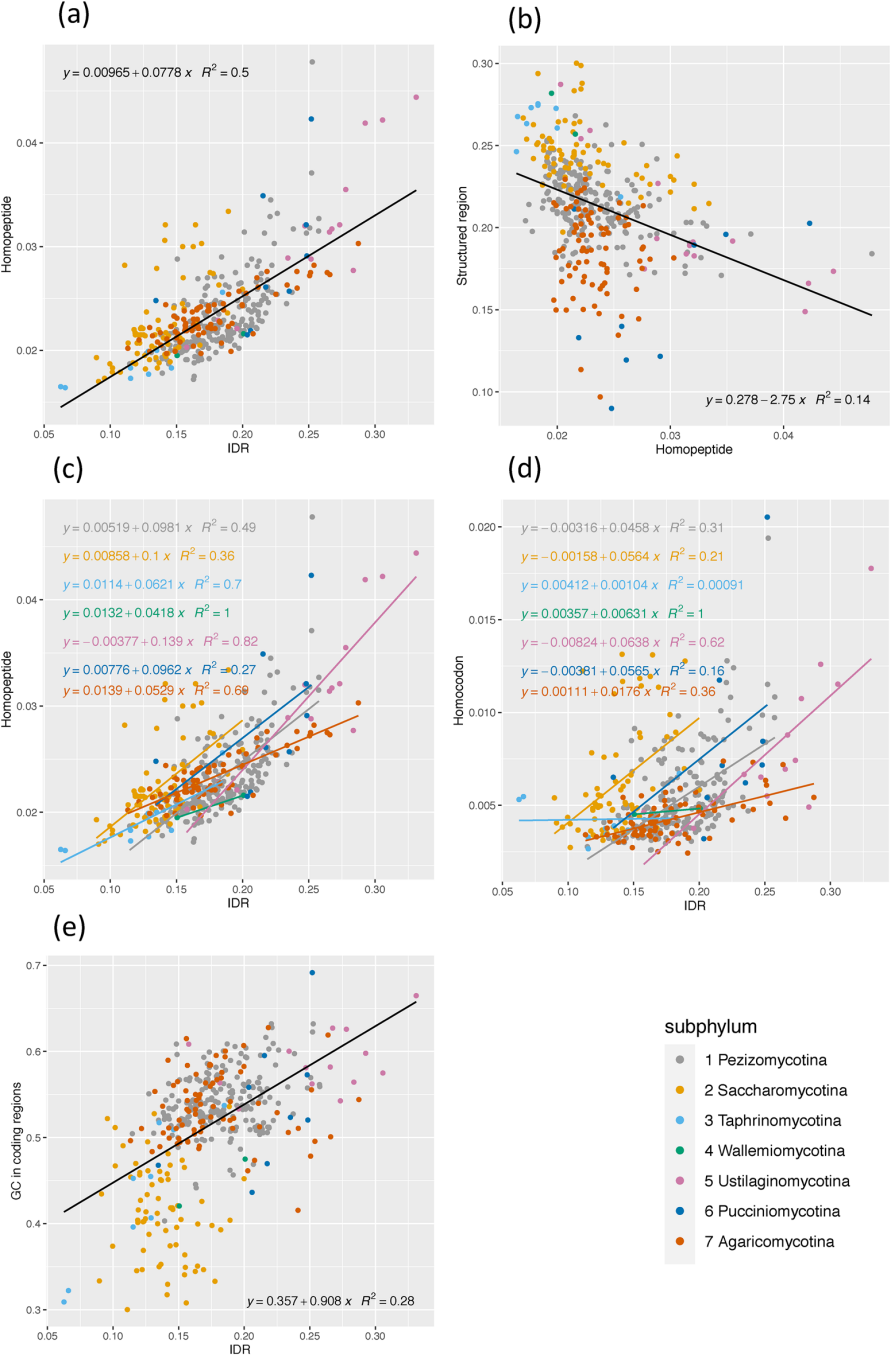
Relationship of homopeptide/homocodon fractions with intrinsic disorder, structured domains and GC content. Scatter plots are drawn of: (**a**) homopeptide fraction versus annotated IDR fraction, with an overall linear regression fitted (*P* value < 0.00001). (**b**) homopeptide fraction versus fraction of structured domains, with an overall linear regression (*P* value < 0.00001). (**c**) homopeptide fraction versus annotated IDR fraction, with linear regressions fitted for each subphylum. *P* values for correlations are < 0.05, except for *Wallemiomycotina*. (**d**) homocodon fraction versus annotated IDR fraction, with regressions for each subphylum (correlation *P* values are < 0.05, except for *Wallemio-*,* Taphrino-* and *Pucciniomycotina*). (**e**) GC fraction in coding regions versus annotated proteome IDR fraction (*P* value < 0.00001).

Furthermore, homopeptide fraction is significantly correlated with annotated IDR fraction across *Dikarya* and also within each subphylum (Fig. 6a,c), but anti-correlated with structured protein-domain content in proteomes (Fig. 6b). The large AT-rich sub-phylum *Saccharomycotina* has less correlation than GC-rich sub-phyla generally, maybe because of the favouring of GC-richness in IDRs (Fig. 6c, and see below for Fig. 6e). This result builds on previous observations on diverse cellular organisms that IDRs evolve along with homopeptide expansion^19, 20^. Although homopeptides are also common in structured regions, total homopeptide lengths mostly vary in IDRs, and homopeptide abundance largely affects the size of IDRs but not of structured regions. Indeed, IDRs generally have higher insertion/deletion rates, and intrinsic disorder content is the major determinant of protein length^23, 47–49^. Also, the general prevalence of the amino-acid types in homopeptides is mirrored by their prevalences in annotated IDRs (save for hydrophobic residues, particularly leucine and valine) (Suppl. Figure S4).

Previous research found that GC-richness is linked to increased proteomic intrinsic disorder^50, 51^. Here, GC level and IDR fraction have significant positive correlation, but not to the same extent as for homopeptide levels *versus* IDR fractions; also, AT-biased genomes, especially *Saccharomycotina*, deviate more from the regression line (Fig. 6e). Indeed, 4 out of the 10 most common amino acids in homopeptides within annotated intrinsic disorder have GC-biased codon repertoires (P, A, G, R), five have AT/GC-even repertoires (S, E, D, Q, T), and only one AT-rich (K) (Figure S4D-E). Although homocodon fraction also positively correlates with IDR fraction, this is less than the correlation between homopeptides and IDRs (Fig. 6a,d), indicating that homocodons are less characteristic of IDRs. However, some sub-phyla are relatively more correlated indicating more homocodon content in their IDRs.

Two algorithms were used to annotate IDRs. IDRs rich in some amino acids might be underestimated, e.g., asparagine, considering its hydrophilicity and enrichment in *S. cerevisiae* prion domains, which have intrinsic disorder^52–55^. If so, IDR and homopeptide fractions (Fig. 6a) would be more correlated, and the correlation of IDR and GC level would be less (Fig. 6e).

## Conclusions

Here we examined the diverse, well-sampled fungal sub-kingdom *Dikarya* for trends in the variation of homopeptides. The *Dikarya* fungi are particularly attractive for such analysis (as explained in full in “Methods” section), not least because they comprise large clades made from AT- and GC-biased species. We observed that amino-acid homopeptide frequencies vary diversely between clades (even between closely related organisms), with the AT-rich *Saccharomycotina* trending distinctly. Dissection of this variation has yielded multiple insights, including:

1. *Homopeptides tend to be GC-rich even for AT-rich genomes*,* indicating they absorb AT bias less or are inherently more GC-rich.* This trend is less pronounced for homocodons. We surmised that these tendencies may be because GC level is easier to increase in homocodons/homopeptides than AT-level, owing to several factors including inherent slippage rates of individual trinucleotides such as CAG/GTC and CGG/GCC.
2. *Homocodon/homopeptide accumulation is strongly coupled to GC/AT bias*,* with a dual bi-furcated correlation between homocodon/homopeptide levels and GC or AT bias.* This indicates that mid-GC species tend to have fewer homocodons/homopeptides simply because they are mid-GC.
3. *Homocodon codon preferences are correlated with AT/GC bias for some codons*,* but not for others.* When homocodon codon preferences were examined for the amino acids encoded by two alternative codons, we found that while some amino-acid codon choices follow genomic AT/GC bias trends (e.g., Glu), others do not (e.g., Lys). Again, we surmise that this is due to different inherent slippage rates for different codons during DNA replication and recombination.
4. *The purity of homopeptides (*i.e.,* the degree to which they are encoded by one specific codon) is modulated by GC/AT bias.* The amino acids that vary the least in homopeptide purity have codon repertoires that are balanced for A + T and G + C. Homopeptide codon usage is most volatile for poly-Arg which has only one AT-biased codon (out of six), presumably because response to an AT-biasing mutational trend or selection pressure is largely dependent on mutation to one codon, whereas five are available for the opposite trend/pressure.
5. *Intrinsic disorder is correlated with both homopeptide frequency and variability across* Dikarya,* but is less correlated for the AT-rich* Saccharomycotina*.* Also, we observe an opposing correlation and anti-correlation with homopeptide levels for intrinsic disorder and structured domains respectively; this (anti-)correlation pair may be capturing a signal from increased IDR insertion/deletion rates^23, 47–49^. Some sub-phyla have homocodon levels relatively more correlated with IDR content, indicating more homocodon content in their IDRs.
6. *Despite the overall trends involving GC/AT bias and intrinsic disorder*,* some amino acids have unique behaviours.* For example, polyglutamine levels are highly variable across *Dikarya*, yet they are encoded by a GC/AT-balanced codon repertoire (CAG/CAA). We suggest that this variability is linked to glutamine preferring to exist in IDRs, which are under less structural constraints^56^, combined with its codon CAG being one of the codons most prone to DNA slippage during replication^36^. For lysine (codons: AAG/AAA), the predominant codon overwhelmingly tends to AAG in homocodons; we hypothesize that this may also be due to inherent lack of slippage ability during DNA replication for the AAA codon. Also, arginine (codons: AGA/AGG/CGT/CGC/CGA/CGG) demonstrates high homopeptide purity in the AT-rich *Saccharomycotina* owing to it having only one AT-rich codon.

## Methods

### Proteome data

In total, 405 *Dikarya* reference proteomes (and corresponding coding regions) were downloaded from UniProt (www.uniprot.org) in July 2018^57^. *Dikarya* provide a good set for analyzing the principles and trends of proteome evolution, since they are comprised of the two main currently well-sampled fungal phyla (*Ascomycota* and *Basidiomycota*), that contain hundreds of fungi of interest as pathogens, and useful for food, biotechnology and laboratory research. Also, there are currently major genome-sequencing initiatives underway to improve further the sampling of the phylogenetic tree of *Saccharomycotina* (the Y1000 + project^58^), and of fungi generally (the 1000 Fungal Genomes project^59^). Furthermore, our previous work on the evolution of prion and prion-like proteins which motivated the present study was focused on fungi^27^. They also contain large clades that are made from either AT- or GC-biased genomes^27^.

### *Dikarya* phylogenetic analysis

*Dikarya* phylogenies were built from 18 s rRNA gene sequences, which are a prominent fungal phylogenetic marker^60^. The multiple sequence alignment (MSA) of the 18S rRNA gene was obtained from SILVA^61^ in March 2018, and reduced to the 405 *Dikarya* reference species. Based on the MSA, phylogenetic trees were made with the maximum likelihood phylogeny program PhyML 3.0^62^, using aBayes branch support and defaults for nucleotide sequences. Trees and associated data were depicted with ggplot2^63^ and ggtree^64^.

### Homopeptide and homocodon frequencies

**Figure 7:**
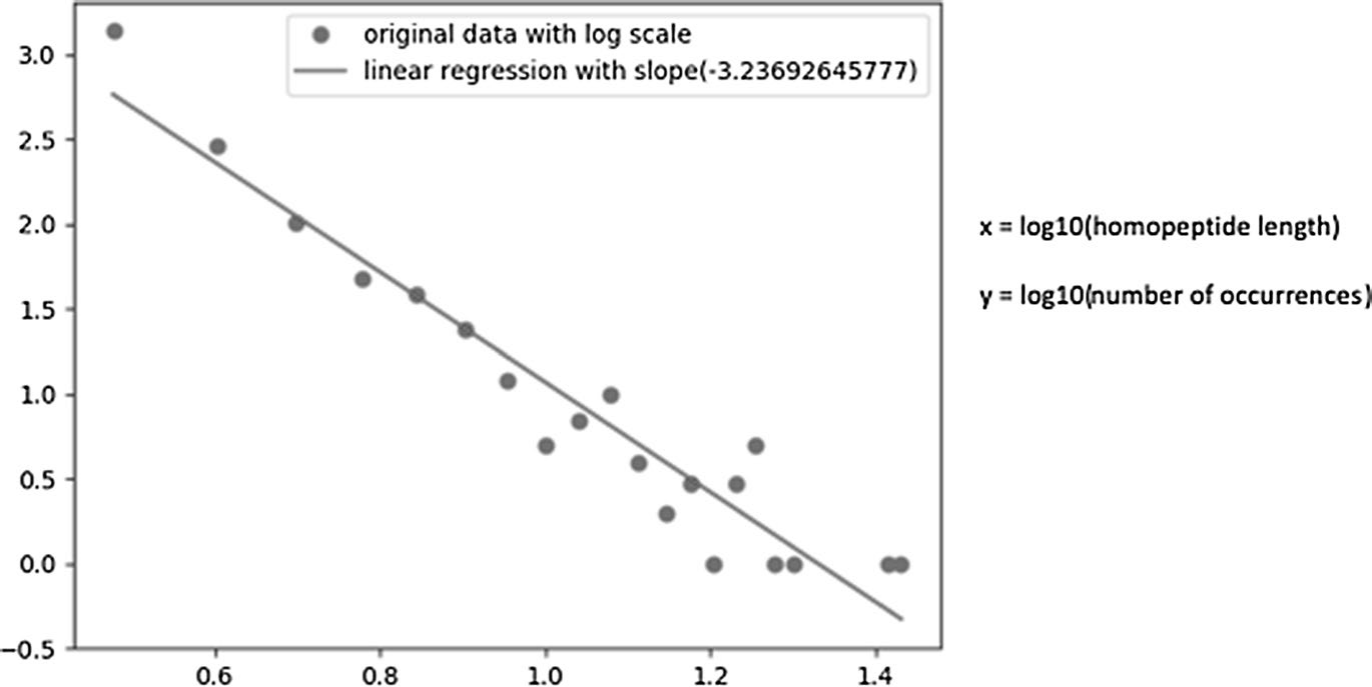
Example of a log–log plot used in the analysis of homopeptide or homocodon distributions. The length distributions are analyzed as log–log scale plots of the number of occurrences of a given homopeptide length versus homopeptide length. The distributions are characterized as linear regressions, yielding a calculated power-law relationship between homopeptide length and frequency for a given amino-acid type.

Homopeptides or homocodons were defined as runs of consecutive single amino acids or codons respectively. In this study, the minimum length of homopeptides and homocodons is three, and only homocodons in coding regions were considered. The positions and lengths of homopeptides were found and calculated for each proteome. The length distributions of homopeptides were further calculated in log scale and made into log–log scatter plots for each of the 10 most abundant amino acids in homopeptides (for example, Fig. 7). The slopes of linear regressions were used to indicate the general quantitative distributions of the homopeptides, i.e., a steeper slope indicated a greater relative amount of short homopeptides in the proteome. The length distributions for the twenty most abundant homocodons were calculated in the same way as for homopeptides. Within each proteome, the types of amino acid were ranked according to their frequencies of homopeptides to give *frequency ranks*, i.e., rank 1 for the most frequent amino-acid homopeptide, rank 2 for the next, etc. Mean frequency ranks (and standard deviations of frequency rank) were calculated for each amino-acid type across *Dikarya* and *Saccharomycotina* to show the variation in the frequencies of homopeptides made from these amino acids (Table 1). Similar rankings were made for homocodon codons.

### Homopeptide purity

A homopeptide could be composed of different codons encoding the same amino acid. To measure the extent to which homopeptides are encoded by a predominant codon, we calculated the ‘purity’ of homopeptides for each type of amino acid X using the equation below:

$${purity}_{aa}= \frac{\sum n}{N}$$

with the counts given by: n = number of the predominant (most frequent) codons in one X-homopeptide, N = number of codons in all X-homopeptides.

The purity of each amino acid is further scaled through dividing by the maximum purity across the 405 proteomes for amino acids with equal codon numbers. However, those encoded by codons with six-fold degeneracy will be generally less pure than those encoded by codons with less degeneracy. Thus, only the overall variance of purity is comparable between different amino acid types (in Table 1).

### Intrinsic disorder

Intrinsically disordered regions (IDRs) in proteomes were annotated by the default DisoPred3 and IUPred2A programs^65, 66^. Many IDR annotators are only available as webservers, so cannot be used here. IUPred and DisoPred are available standalone and were ranked in the top three in at least one assessment^67^. Combined use of multiple such programs improves annotation^68^. Only IDRs ≥ 30 residues long were considered, since typically an IDR of ≥ 30 residues is classified as a ‘long’, about a third of eukaryotic proteins have such long IDRs, and programs trained on long IDRs are less accurate for shorter IDRs^68^. We used the union set of IUPred and DisoPred results after comparing the differences in their annotation, since we did not want to be restricted by any tendency of a program to under-annotate IDRs with specific compositional traits. In total, only 5.6% of DisoPred results are not predicted by IUPred with a proximity threshold of 10 amino acids; 20.15% of IUPred prediction are not predicted by DisoPred.

A scale of the propensity of amino-acid types to favour disorder or structure was calculated. The fractions of each amino-acid type were derived for an IDR set from the DISPROT database^52^ (version 7.0, reduced for redundancy as previously described^69^), and from the ASTRALSCOP40 protein domain database^70^ (version 2.06). For the latter, the sequences derived from the Protein Data Bank file atom records were used, to minimize inclusion of intrinsic disorder. The fractions for each amino acid in the DISPROT set were then divided by the corresponding fractions in ASTRALSCOP. The logarithm of this ratio was calculated to make a propensity (termed **P**_**diso**_) that is positive for amino acids favouring disorder and negative for those favouring structure. Table 1 lists the scale.

### Structured domain annotations

Annotations of structured domains were made by mapping the ASTRALSCOP95 data set^70^ onto proteomes using BLASTP (e-value threshold = 0.0001)^71^. Blast matches were sorted on increasing order of e-value, and progressively de-selected from the list if they overlap a match of smaller e-value.

## Supplementary information

Supplementary Informations.

## Data Availability

The data analyzed are publicly available from the Uniprot^57^, SILVA^61^, DISPROT^69^, and ASTRALSCOP databases^70^. Some generated data is available in Table 1 and in the Supplementary Information. Other generated data is available from the authors upon request.
