# Supplementary material for: Homopeptide and homocodon levels across fungi are coupled to GC/AT-bias and intrinsic disorder, with unique behaviours for some amino acids
Source: Sci Rep. 2021 May 11;11:10025. doi: 10.1038/s41598-021-89650-1 (PMC8113271; doi:10.1038/s41598-021-89650-1)
Supplement: Supplementary file 1 — Supplementary Informations. [file 41598_2021_89650_MOESM1_ESM.pdf]

Supplementary File for:

Homopeptide and homocodon levels across fungi are coupled to GC/AT-bias and intrinsic disorder, with unique behaviours for some amino acids

Yue Wang[1] and Paul M. Harrison[1] *

[1] Dept. of Biology,

McGill University,

Montreal, QC, Canada.

* corresponding author: paul.harrison@mcgill.ca

Figure S1: The phylogeny of *Ascomycota* and *Basidiomycota* in detail, showing the coloured sections c and d annotated with amino-acid types and codon types, and with individual species shown. The annotations are in four sections *a, b, c* and *d*. Sections *a* and *b* show the overall homopeptide and IDR fractions as in Figure 2 (but in that figure the mean values are shown). At section *c*, are the log-log plot slopes of length distributions of top 10 most frequent amino-acid types in homopeptides, ranked in decreasing order of abundance. The names of the top 10 amino acids types are shown. The method of log-log distribution plot making is described in *Materials and Methods*. At section *d* are log-log plot slopes of length distributions of the top 20 frequent codon types in homocodons, ranked in decreasing order of abundance. The codon names are shown. The key to these sections is shown below. For sections *a* and *b* the colour spectra show the log of the homopeptide and IDR fractions respectively. For sections *c* and *d*, the colour spectra show the slope of the line fitted to the length distributions on a log-log plot, as described in *Methods*. The data for *Ascomycota* and *Basidiomycota* are shown on following separate pages. The position of the subphylum *Saccharomycotina* and the genus *Saccharomyces* are labelled.

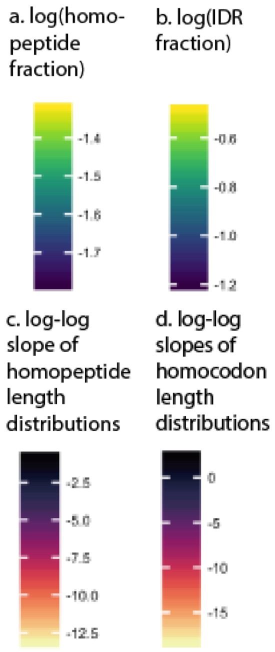

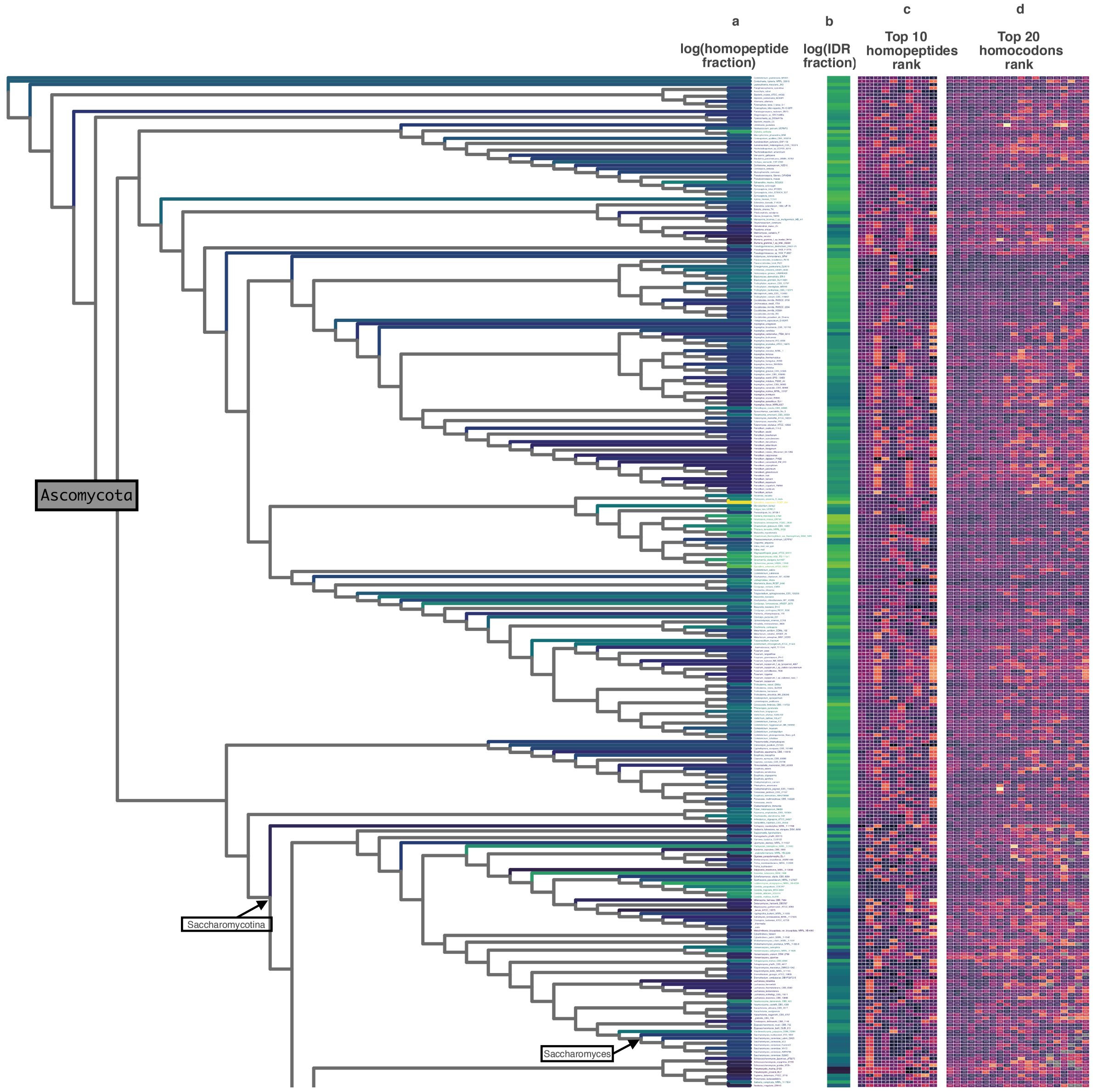
a
log(homopeptide fraction)
b
log(IDR fraction)
c
Top 10 homopeptides rank
d
Top 20 homocodons rank
Ascomycota
Saccharomycotina
Saccharomyces

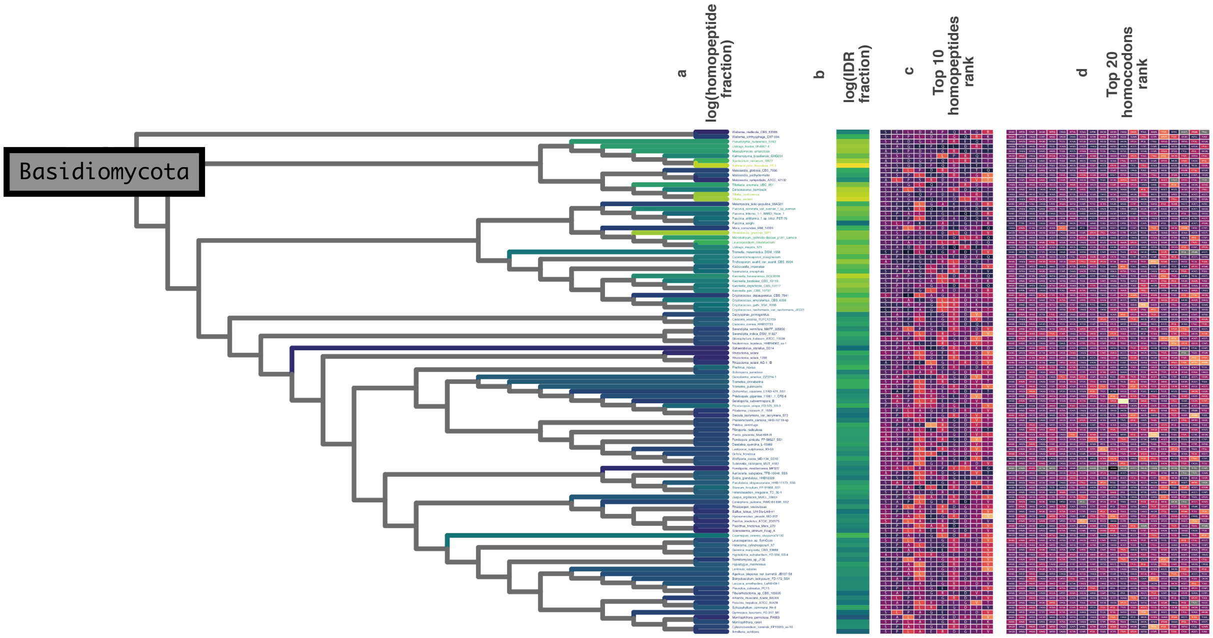

Basidiomycota
a
log(homopeptide fraction)
b
log(IDR fraction)
c
Top 10 homopeptides rank
d
Top 20 homocodons rank

Figure S2: The relationship between homopeptide length trends and frequencies.

(A) The residue that exhibits the strongest relationship between its homopeptide log(length) distribution slope and frequency is glycine (G).

(B) A table of the correlations between the homopeptide log(length) distribution slope and frequency for all of the amino acids.

(A)

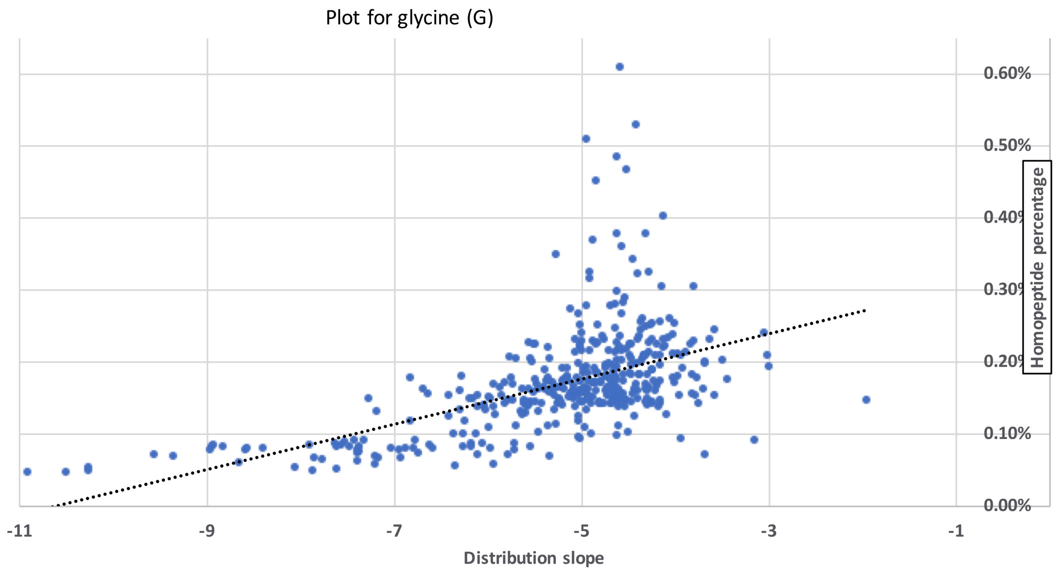

(B)

Amino Acid	Correlation coefficient (R)	P-value	
significant P-values for correlation			
G	0.525	<0.00001	**shown in (A)**
T	0.331	<0.00001	
D	0.271	<0.00001	
Q	0.239	<0.00001	
F	0.224	<0.00001	
C	0.176	0.00038	
S	0.174	0.0004	
M	0.167	0.0073	
N	0.157	0.00158	
E	0.136	0.0063	
V	0.117	0.0181	
A	0.11	0.028	
no significant P-values			
H	0.097	NS	
Y	0.095	NS	
L	0.092	NS	
R	0.05	NS	
W	0.044	NS	
I	0.014	NS	
significant P-values for anti-correlation			
P	-0.104	0.037	
K	-0.157	0.00158	

Figure S3: Examples of the predominant codon for amino acids with two alternative codons switching according to AT/GC-level across organisms. (a) Poly-GAG(E) to poly-GAA(E) ratio versus GC (as a fraction of 1.0). (b) Poly-GAC(D) to poly-GAT(D) ratio versus GC (as a fraction of 1.0).

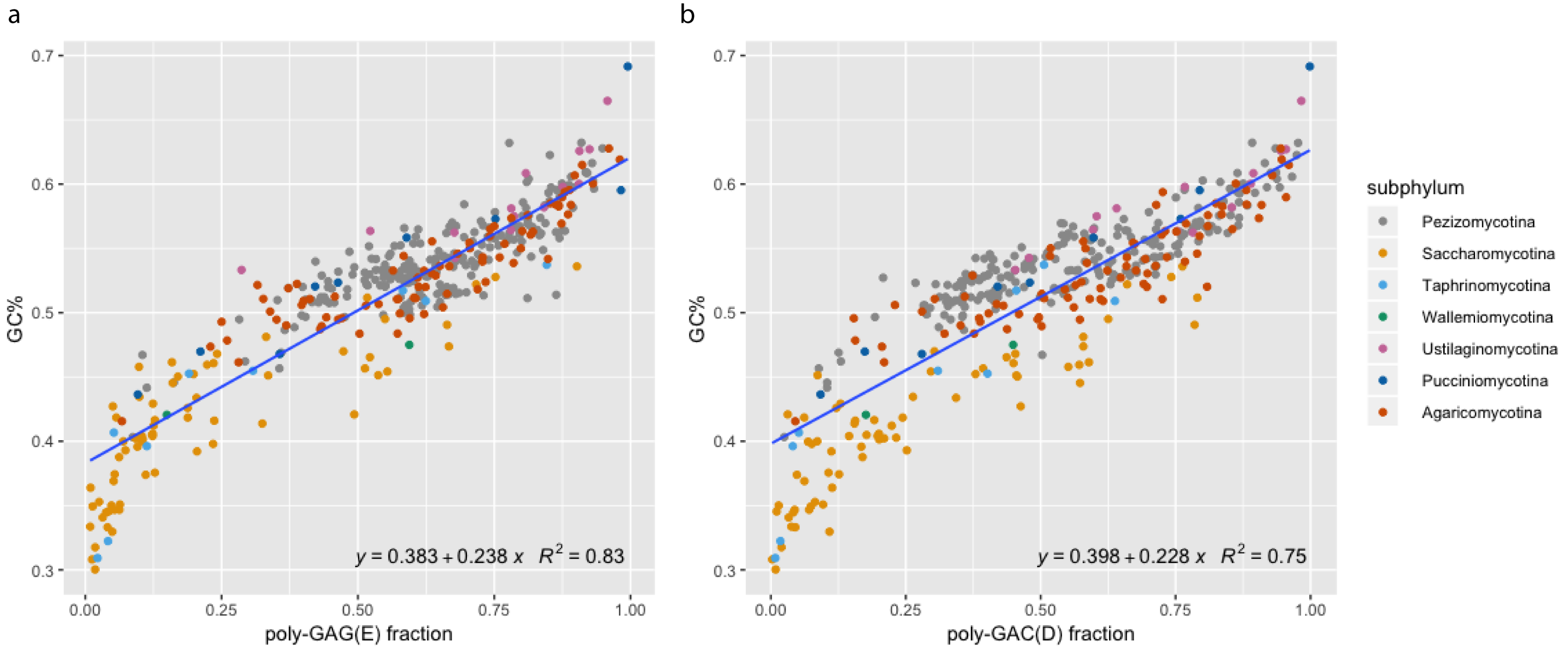
a
b
GC%
0.7
0.6
0.5
0.4
0.3
0.00
0.25
0.50
0.75
1.00
poly-GAG(E) fraction
poly-GAC(D) fraction
y = 0.383 + 0.238 x   R2 = 0.83
y = 0.398 + 0.228 x   R2 = 0.75
subphylum
Pezizomycotina
Saccharomycotina
Taphrinomycotina
Wallemiomycotina
Ustilaginomycotina
Pucciniomycotina
Agaricomycotina

Figure S4: Homopeptide and amino acid usage in proteomes. (a) Overall proteome usage of each amino acid; (b) ratio of homopeptide amino acids to non-consecutive single amino acids; (c) the fraction of each amino acid type in homopeptides; fraction of each amino acid in homopeptides in IDRs annotated with (d) IUPred and (e) DisoPred.

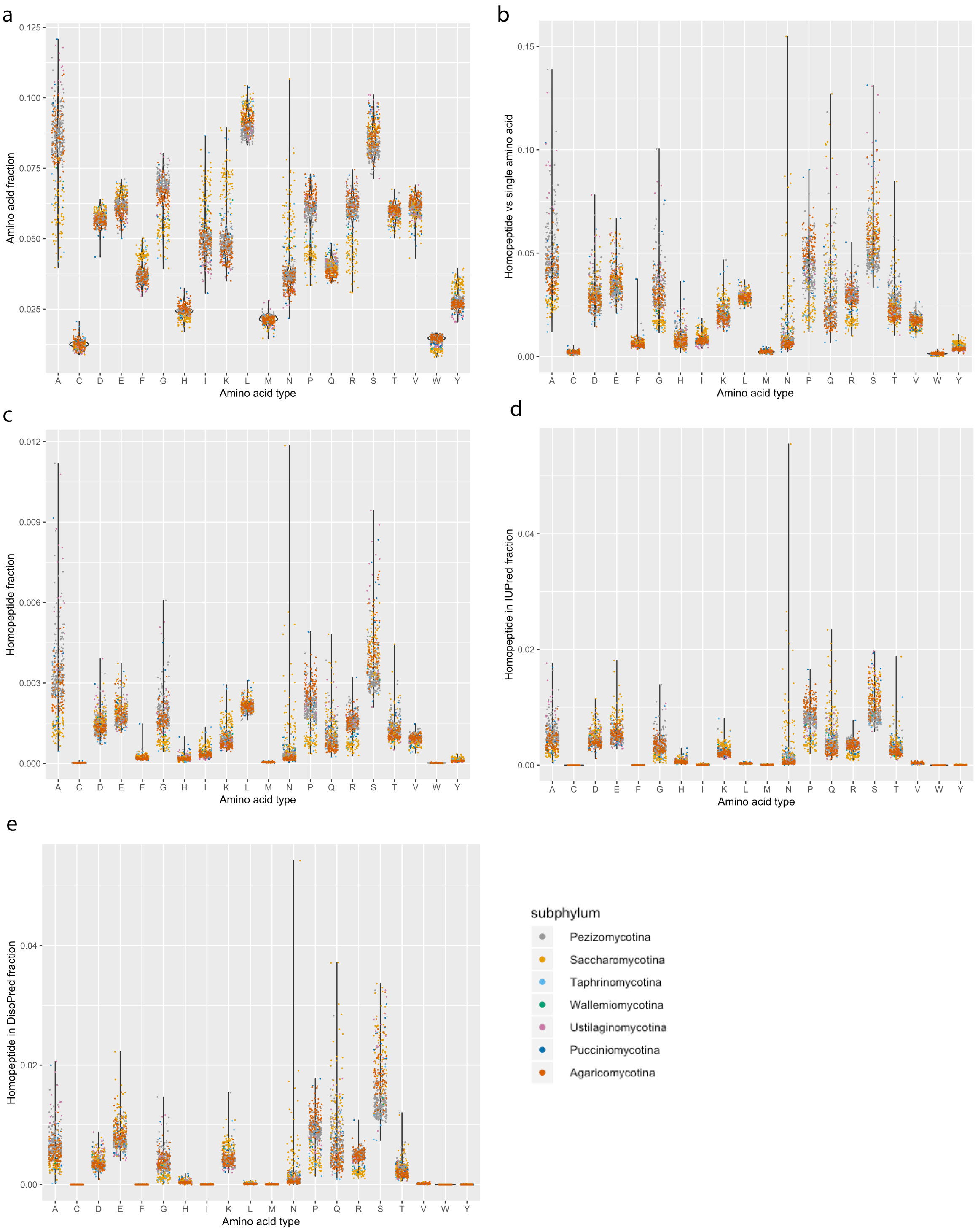

a
Amino acid fraction
0.125
0.100
0.075
0.050
0.025
A C D E F G H I K L M N P Q R S T V W Y
Amino acid type
b
Homopeptide vs single amino acid
0.15
0.10
0.05
0.00
A C D E F G H I K L M N P Q R S T V W Y
Amino acid type
c
Homopeptide fraction
0.012
0.009
0.006
0.003
0.000
A C D E F G H I K L M N P Q R S T V W Y
Amino acid type
d
Homopeptide in IUPred fraction
0.04
0.02
0.00
A C D E F G H I K L M N P Q R S T V W Y
Amino acid type
e
Homopeptide in DisoPred fraction
0.04
0.02
0.00
A C D E F G H I K L M N P Q R S T V W Y
Amino acid type
subphylum
Pezizomycotina
Saccharomycotina
Taphrinomycotina
Wallemiomycotina
Ustilaginomycotina
Pucciniomycotina
Agaricomycotina

Table S1: Means and standard deviations of purities for homopeptides of the individual amino-acid types in subphyla and classes. Homopeptide purities of each amino acid except W and M grouped as follows: VIL (aliphatic hydrophobic); FYH (aromatic); DE (negative charge); KR (positive charge); QN (prion-linked); GA (small); ST (hydroxyl), P and C.

Supplementary Table: Means and standard deviations of purities for homopeptides of the individual amino-acid types in subphyla and classes *

	Aliphatic hydrophobic			Aromatic			Negatively charged		Positively charged		Prion-linked		Small		Hydroxyl			
**Means of purities**	**V**	**I**	**L**	**F**	**Y**	**H**	**D**	**E**	**K**	**R**	**Q**	**N**	**G**	**A**	**S**	**T**	**P**	**C**
**Pezizomycotina**	0.714	0.790	0.626	0.829	0.812	0.775	0.765	0.762	0.845	0.612	0.767	0.829	0.721	0.701	0.609	0.708	0.662	0.799
Dothideomycetes (34)	*0.704*	*0.820*	*0.631*	***0.869***	*0.832*	*0.784*	*0.762*	*0.759*	*0.872*	*0.616*	*0.766*	*0.853*	*0.707*	*0.697*	*0.607*	*0.701*	*0.660*	*0.795*
Leotiomycetes (16)	*0.690*	*0.726*	*0.609*	*0.803*	*0.785*	*0.784*	*0.775*	*0.763*	*0.809*	*0.619*	*0.754*	*0.791*	*0.686*	*0.695*	*0.587*	*0.681*	*0.672*	*0.775*
Eurotiomycetes (90)	*0.700*	*0.769*	*0.607*	*0.812*	*0.793*	*0.764*	*0.754*	*0.750*	*0.818*	*0.597*	*0.756*	*0.808*	*0.705*	*0.686*	*0.594*	*0.698*	*0.648*	*0.785*
Sordariomycetes (73)	*0.742*	*0.820*	*0.651*	*0.841*	*0.834*	*0.784*	*0.778*	*0.779*	***0.876***	*0.626*	*0.785*	*0.855*	*0.757*	*0.725*	*0.634*	*0.732*	*0.679*	*0.825*
**Saccharomycotina (69)**	0.739	0.711	0.628	0.746	0.784	0.778	0.786	0.790	0.757	**0.744**	0.759	0.786	**0.790**	**0.754**	0.602	0.709	**0.729**	0.784
**Taphrinomycotina (9)**	0.723	0.751	0.620	0.788	0.808	0.803	0.801	0.780	0.793	0.630	0.758	0.797	0.738	0.743	0.610	0.694	0.713	0.764
**Ustilaginomycotina (14)**	0.767	**0.879**	**0.668**	0.834	**0.869**	0.782	0.773	0.768	0.864	0.623	0.791	**0.881**	0.728	0.697	0.638	0.765	0.677	**0.845**
**Pucciniomycotina (9)**	**0.772**	0.838	0.655	0.834	0.841	**0.826**	**0.808**	**0.805**	0.830	0.636	**0.803**	0.858	0.730	0.732	**0.648**	**0.774**	0.690	0.818
**Agaricomycotina (80)**	0.701	0.763	0.610	0.817	0.787	0.778	0.772	0.753	0.807	0.606	0.769	0.810	0.681	0.688	0.592	0.695	0.651	0.794
**Standard Deviations of purities**	**V**	**I**	**L**	**F**	**Y**	**H**	**D**	**E**	**K**	**R**	**Q**	**N**	**G**	**A**	**S**	**T**	**P**	**C**
**Pezizomycotina**	0.034	0.048	0.035	0.036	0.036	0.027	0.022	0.021	0.041	0.026	0.025	0.039	0.038	0.029	0.034	0.038	0.022	0.042
Dothideomycetes (34)	*0.030*	*0.053*	*0.036*	*0.042*	*0.038*	*0.030*	*0.020*	*0.017*	*0.036*	*0.031*	*0.022*	*0.042*	*0.033*	*0.029*	*0.029*	*0.042*	*0.018*	*0.037*
Leotiomycetes (16)	*0.021*	*0.029*	*0.027*	*0.036*	*0.023*	*0.021*	*0.009*	*0.015*	*0.043*	*0.022*	*0.019*	*0.011*	*0.014*	*0.012*	*0.020*	*0.023*	*0.013*	*0.037*
Eurotiomycetes (90)	*0.026*	*0.045*	*0.031*	*0.031*	*0.030*	*0.022*	*0.019*	*0.019*	*0.035*	*0.027*	*0.018*	*0.038*	*0.032*	*0.029*	*0.035*	*0.036*	*0.024*	*0.040*
Sordariomycetes (73)	*0.040*	*0.036*	*0.038*	*0.030*	*0.039*	*0.030*	*0.027*	*0.025*	*0.037*	*0.026*	*0.032*	*0.036*	*0.040*	*0.030*	*0.036*	*0.038*	*0.020*	*0.047*
**Saccharomycotina (69)**	0.059	0.032	0.057	0.034	0.042	0.041	0.042	0.040	0.041	**0.103**	0.042	0.028	0.067	0.055	0.034	0.039	0.049	0.058
**Taphrinomycotina (9)**	**0.093**	**0.103**	0.075	0.055	0.064	0.035	0.048	0.025	0.052	0.038	0.049	0.065	0.050	0.032	0.040	0.071	0.029	0.082
**Ustilaginomycotina (14)**	0.082	0.080	**0.089**	0.069	0.059	0.055	0.056	0.061	0.090	0.042	0.057	0.057	0.050	0.043	0.050	0.049	0.039	0.052

**Pucciniomycotina (9)**	0.041	0.065	0.055	0.067	0.064	0.037	0.034	0.052	0.075	0.045	0.054	0.061	0.032	0.021	0.025	0.029	0.021	0.058
**Agaricomycotina (80)**	0.082	0.094	0.072	**0.101**	**0.094**	**0.091**	**0.089**	**0.088**	**0.098**	0.065	**0.091**	**0.097**	**0.076**	**0.078**	**0.069**	**0.080**	**0.074**	**0.102**

* Maxima in each column for either mean or standard deviation are in bold. The number of species in a clade is in brackets after the clade name. The column for arginine is highlighted in red as it is specifically discussed in the text.
